# Validation of the Utrecht work engagement scale (UWES-9) in the Czech Republic

**DOI:** 10.1038/s41598-025-26907-z

**Published:** 2025-11-28

**Authors:** Martin Heveri, Lukas Novak, Iva Polackova Solcova, Peter Tavel

**Affiliations:** 1 Olomouc University Social Health Institute, Palacký University in Olomouc, Univerzitní 244/22, 771 11 Olomouc, Czech Republic; 2 Institute of Psychology, The Czech Academy of Sciences, Prague, Czech Republic

**Keywords:** Work engagement, Validity, Extraversion, Neuroticism, Self-efficacy, Risk factors, Psychology, Human behaviour, Occupational health

## Abstract

The Utrecht Work Engagement Scale (UWES) is the most widely used work engagement measure. However, its cultural validity regarding factor structure and other psychometric characteristics remains unclear in certain cultural contexts. Thus, this study aimed to assess UWES-9, shortened version of UWES, in the Czech cultural environment psychometrically. A Sample of 707 employees (Age: *M* = 43.65, *SD* = 10.08, Females: 38.47%) recruited from various companies in the Czech Republic was used. Neuroticism, extraversion, self-efficacy, spirituality, chronic health diseases, and frequency of health risk behavior were measured. Higher UWES-9 total scores were reported for professional staff, managers, and people with higher vocational school or university scores. Additionally, a significant difference was also found in family status, with individuals in a relationship reporting higher work absorption. Among the tested factor models the modified three-factor solution: χ^2^ (20) = 158.853; *p* < .0.001; CFI = 0.987; TLI = 0.976; RMSEA = 0.077; SRMR = 0.017 reached the highest fit with the data. Measurement equivalence testing suggested that on configural, metric, scalar, and strict levels, the UWES-9 assesses work engagement invariantly between genders. A positive association with extraversion, self-efficacy, and a negative association with neuroticism supported the convergent validity of the UWES-9. Internal consistency (α = 0.96, McDonald’s ω = 0.96) was excellent. Logistic regression revealed that a higher score in the UWES-9 was associated with a lower chance of developing skin diseases and pain of unclear origin. The cultural validity of the UWES-9 in terms of factor structure was supported. The UWES-9 is a valid and reliable instrument to measure work engagement in the Czech cultural context.

**Supplementary Information:**

The online version contains supplementary material available at 10.1038/s41598-025-26907-z.

## Introduction

In recent years, we have witnessed an increasing influence of positive psychology, which focuses on “positive” behaviour, performance, and well-being rather than the study of dysfunctional aspects of behaviour, cognition, and emotion^1^. In occupational health psychology, new concepts of employees’ organisational behaviour^2^ are linked to employees’ positive work experiences. One of the most important concepts is Work engagement (WE).

The WE is defined as “a positive, fulfilling, work-related state of mind that is characterised by vigor, dedication, and absorption”^3^. The vigor dimension means high levels of energy and mental resilience while working, the willingness to invest effort in one’s work, and persistence even in the face of difficulties. The second one, dedication reflects significance, enthusiasm, inspiration, and pride in connection with one’s work. The third dimension, absorption, refers to full concentration and being happily engrossed in one’s work, whereby time passes quickly, and detachment is difficult^3^.

Based on this conceptualisation of WE, a self-report measurement tool - Utrecht Work Engagement Scale (UWES) - was created. Initially, authors of the UWES, Schaufeli and Bakker^4^, introduced a questionnaire consisting of 17 items called the UWES-17. The three dimensions are structured in the following way: vigor—measured by six items, dedication—by five items, and absorption—by six items. Later, Schaufeli et al.^5^ created a shortened version—UWES-9, consisting of nine items, each dimension comprising three items each. There is also an ultra-short three-item version of the UWES available, which covers each of the three dimensions of WE with one item^6^. The UWES has been translated into more than 30 languages^7^, and its psychometric properties were examined in employee groups from more than 30 countries. More recent ones include: Serbia^8^, Lithuania^9^, or Vietnam^10^.

There are several alternative measures of WE available, e.g., Job Engagement Scale^11^, the Intellectual, Social, Affective Engagement Scale—ISA Engagement Scale—^12^, Shirom-Melamed Vigor Measure—SMVM -^13^, and others. We have decided to explore the psychometric features of the UWES because it is the most widely used instrument to measure WE assessment^14–16^. Its psychometric parameters have been tested in various cultures, employment groups, international studies, and longitudinal studies. However, despite its wide use and relatively sound psychometric properties, its factorial validity in different cultural contexts remains ambiguous. For instance, studies^17–20^ conducted in different cultural environments than the original validation UWES study indicated that the factor structure was different. This questions the cultural validity of the UWES-9 in terms of factor structure.

To explore the cultural validity of the UWES in terms of factor structure in different cultural environments, we examined its psychometric properties on the sample from the Czech Republic. We have chosen to examine psychometric parameters of the shortened UWES, 9-item version, for the following reasons: first, this shortened version is considered more valid and reliable as compared with the original version^21^; second, it is more convenient for use in the large test batteries and reduces the attrition likelihood due to fewer items^5^.

Along with the cultural validity testing, we also aimed to investigate convergent validity. In particular, we explored whether the UWES-9 scores were associated with personality characteristics (neuroticism and extroversion), self-efficacy, and health-related outcomes. More detailed rationale for including these constructs in the present study is as follows:

Personality traits play an important role in shaping work engagement. The Five-Factor Model conceptualizes personality in terms of five broad domains: neuroticism, extraversion, openness, agreeableness, and conscientiousness^22^. In the present study, we focused on two core traits consistently related to engagement: neuroticism, which reflects emotional instability and proneness to distress, and extraversion, which reflects sociability, assertiveness, and positive affect. Prior findings suggest that higher extraversion and lower neuroticism predict stronger engagement across occupational groups^23,24^.

Another relevant factor for engagement is self-efficacy, defined as the belief in one’s capacity to mobilize cognitive and behavioral resources to meet situational demands^25^. Employees with higher self-efficacy report greater vigor, dedication, and absorption, likely because they perceive challenges as manageable and derive confidence from successful task accomplishment^26^.

Previous studies also highlight the role of physical and mental health in engagement. Chronic health conditions, such as pain or dermatological problems, have been associated with lower work ability and reduced engagement^27^. Engagement, however, may act as a buffer, helping employees maintain energy and performance despite health challenges. In addition, health-risk behaviors (e.g., smoking, alcohol consumption) have been linked to lower engagement, suggesting that lifestyle patterns and well-being are intertwined with work-related motivation. Research indicates that higher engagement predicts better psychological and physical health, lower burnout, and reduced risk of stress-related illness^28^. Conversely, disengagement has been associated with presenteeism and greater vulnerability to chronic conditions.

Finally, we aim to test the discriminant validity of the UWES-9 using spirituality. In the present study, spirituality was assessed using the Daily Spiritual Experience Scale^29,30^, which conceptualizes spirituality as a universal human experience manifesting in everyday life rather than as adherence to religious traditions. It measures the extent to which individuals perceive a connection with the transcendent in daily situations, including awareness of a higher presence, gratitude and interconnectedness, experiences of comfort and strength, and expressions of compassion and love. This form of spirituality emphasizes implicit, ordinary experiences that may be present even in individuals without explicit religious affiliation. We chose spirituality for discriminant validity testing because, while it touches on themes of meaning and purpose, it constitutes a distinct domain not expected to overlap with WE. It was therefore included as a contrasting construct to test discriminant validity, examining whether work engagement can be empirically distinguished from everyday spiritual experiences.

Taken together, the main aim of this study is to explore psychometric properties, especially the reliability and validity of the Czech version of the UWES-9. Specifically, we examined the internal consistency, factorial validity, measurement invariance and construct validity. More specifically, convergent validity was explored by differences in WE according to main sociodemographic characteristics and correlation with self-efficacy, neuroticism, extraversion, chronic health illnesses and health risk behaviors. Lastly, discriminant validity was tested by its relationship to spirituality.

Following previous research findings and theoretical assumptions, we set the hypothesis listed below. The theoretical rationale for these hypotheses is presented in supplementary material online (https://osf.io/w5anh/).

### Hypothesis 1

A three-factor solution of WE would best fit the data.

### Hypothesis 2

The WE would be related to high levels of extraversion (H2a) and low levels of neuroticism (H2b).

### Hypothesis 3

There would be a significant positive association between self-efficacy and the UWES-9 total score.

### Hypothesis 4

The UWES-9 and its subscales would show positive weak correlations with age and managerial work positions.

## Materials and methods

### Participants

The data were collected through the Czech National Panel agency between April 7, 2021, and April 22, 2021. The panel recruits participants through online channels and maintains a large database of respondents. Recruitment follows demographic quotas (based on age, gender, region, and education) to mirror the demographic composition of the Czech population; however, as a non-probabilistic method, it does not ensure full random selection and therefore the sample cannot be considered statistically representative. From the survey (*n* = 1662), participants were excluded who were either without work (*n* = 187), pensioners (*n* = 468), or those who did not answer a question regarding economic status (*n* = 223). This resulted in 784 participants. To increase data quality, we removed subjects finishing the survey quickly, i.e., < 15 min (*n* = 6). The survey typically lasted > 30 min. We also excluded respondents answering discrepantly to quality check items (*n* = 71). These items included information about weight, height, and age. Tolerance in these control questions was set on 2 kg, 2 cm, and 2 years, respectively. After removing these subjects, the final number of participants was 707 (Age: *M* = 43.65, *SD* = 10.08, Females: 38.47%).

## Measures

### Utrecht work engagement scale (UWES)

We used a short version of UWES, UWES-9. The Czech version was obtained from the online repository of the scale author^7^. This version consists of 9 items, of which 3 constitute vigor, 3 of dedication, and 3 of absorption subscales. Items are assessed on a 7-point Likert scale, scoring from 0: “Never” to 6: “Always”. The scale can be scored as a sum of all items, or subscale scores can be created. A higher score indicates a higher degree of WE.

### Daily spiritual experience scale (DSES)

“The Daily Spiritual Experience Scale (DSES) is a 16-item self-report measure designed to assess ordinary experiences of connection with the transcendent in daily life.”^30^. The DSES is validated for the Czech Republic by Malinakova et al.^31^. The Czech version has 15 items and uses a 6-point scale from 1:” Many times a day” to 6: “Never or almost never”. A higher score indicates a higher degree of spirituality. Internal consistency of the DSES was excellent: Cronbach’s $\:\alpha\:$ = 0.96 95% CI [0.95–0.97] and McDonald’s $\:{\omega\:}_{t}$ = 0.96 95% CI [0.95–0.97].

### General self-efficacy scale (GSES)

The General Self-Efficacy Scale (GSES) is a widely used assessment tool developed by Schwarzer and Jerusalem^32^ to measure an individual’s belief in their ability to cope with a wide range of challenging situations and to exert control over their important life events. The Czech version of the GSES^33^ contains 10 items on a 4-point Likert scale—1: “I do not agree”, 2: “I rather not agree”, 3: “I rather agree”, 4: “I agree”. The higher the total score reached, the higher the self-efficacy. Internal consistency of the GSES was excellent: Cronbach’s $\:\alpha\:$ = 0.95 95% CI[0.94–0.95] and McDonald’s $\:{\omega\:}_{t}$ = 0.95 95% CI[0.94–0.95].

### The big five inventory (BFI)

The Big Five Inventory^34,35^ was developed to assess the following personality domains: extraversion, agreeableness, conscientiousness, neuroticism, and openness to experiences. It assesses these domains with 44 short phrases that the respondent answers on a five-point rating scale, ranging from 1: “Strongly Disagree” to 5: “Strongly Agree”^36^. The BFI is validated for the Czech Republic by Hřebíčková et al.^37^. For this study, we used two subscales: (1) Neuroticism (BFI_N) and (2) Extraversion (BFI_E). The neuroticism subscale assesses a tendency to experience negative emotions, such as anger, anxiety, and sadness. Eight items load onto this subscale. The higher the score, the higher the level of neuroticism is identified. Internal consistency of the BFI_N was good: Cronbach’s $\:\alpha\:$ = 0.87 95% CI[0.86–0.89] and McDonald’s $\:{\omega\:}_{t}$ = 0.87 95% CI[0.86–0.89]. The extraversion subscale measures the tendency to interact with people, be enthusiastic, be action-oriented, and be full of energy. The subscale consists of eight statements. Like the Neuroticism scale, a higher score means more extraversion. Internal consistency of the BFI_E was good: Cronbach’s $\:\alpha\:$ = 0.85 95% CI[0.84–0.87] and McDonald’s $\:{\omega\:}_{t}$ = 0.85 95% CI[0.84–0.87].

### Health-risk behaviour

Health-risk behavior was evaluated in the present study using items that specifically addressed behaviors associated with health risks. Participants were asked to respond to the following questions: “In the past month, how often have you…” (1) “Smoked,” (2) “Consumed alcohol,” (3) “Used illegal drugs,” (4) “Consumed coffee,” and (5) “Engaged in recreational screen time (television or computer use).” Responses were recorded on a six-point Likert scale ranging from “never” (1) to “many times a day” (6). For analytical purposes, the frequency of these behaviors was dichotomised: responses indicating engagement “never,” “about once or twice,” or “about every week” were coded as 0 (“Low frequency”), whereas responses indicating engagement “more than once a week,” “every day,” or “many times a day” were coded as 1 (“High frequency”).

### Chronic health complaints

Chronic health complaints were assessed based on the presence of the following conditions: ischemic heart disease, hypertension, stroke/cerebrovascular accident, chronic pulmonary disease, asthma, cancer, diabetes, obesity, arthritis, back pain, gastric or duodenal ulcers, inflammatory bowel diseases (Crohn’s disease, ulcerative colitis), dermatological conditions (e.g., eczema), allergies, migraine, pain of unknown origin, in women—pelvic pain (including gynaecological issues), thyroid disease, gastroesophageal reflux disease (GERD), chronic fatigue, and psoriasis. Participants were asked to indicate whether they had experienced any of these conditions by responding “yes” (1) or “no” (0) to the question: “Have you had any of the following health problems?” with additional response options for “none”.

### Data analysis

Inspection of histograms and results of the Martia test of multivariate skewness and kurtosis indicated that the normality assumption is violated in the UWES-9 items. Moreover, examination of residual plots and the result of the Breusch–Pagan test ($\:chi-squared$ = 7.21, *df* = 1, *p* = 0.007) suggested heteroscedasticity. Examination of the polychoric correlation matrix revealed no extreme multicollinearity among UWES-9 items, with all inter-item correlations below 0.90. Thus, methods not requiring parametric assumptions were used. The Little MCAR test provided evidence that missing values are missing at random. Therefore, as there were a few missing values (*n* = 60), incomplete cases were deleted listwise.

**Fig. 1:**
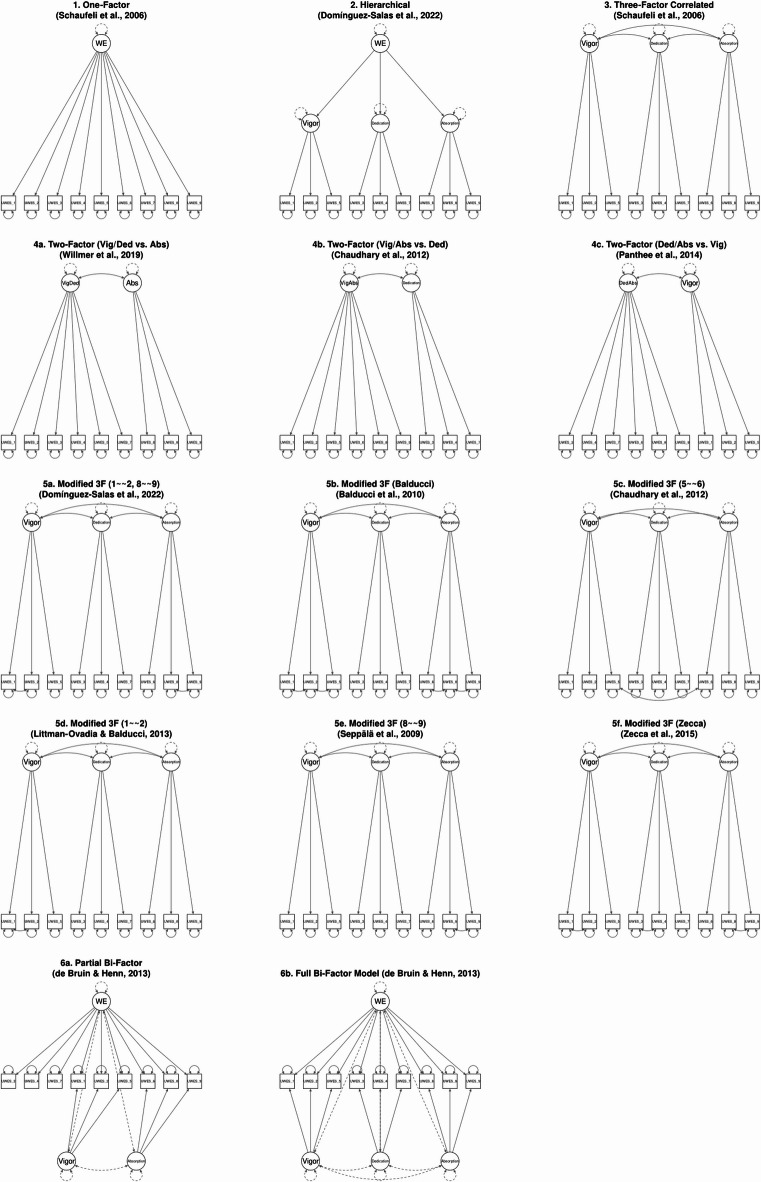
Confirmatory Factor Analysis Models for UWES-9. All 14 unique tested models are displayed with their respective factor structures and correlated errors where applicable.

The instrument’s factor structure was investigated via Confirmatory Factor Analysis (CFA). A series of competing models were specified, reflecting those most prominent in the validation literature. For the full overview and model definitions see Fig. 1 below. The analysis included the original correlated three-factor model (vigor, dedication, absorption) and a unidimensional one-factor model^5^. To account for the consistently high inter-factor correlations, a hierarchical model, with the three factors loading onto a single second-order Work Engagement factor, was also tested^38^. Additionally, several alternative configurations were examined, including various two-factor solutions (e.g., Chaudhary, Rangnekar, & Barua^39^, a partial bi-factor model (de Bruin & Henn^40^, and modified three-factor models with correlated error terms (e.g., Simbula et al.^41^.

Kaiser–Meyer-Olkin (KMO) measure, together with the Bartlett test of sphericity, was applied to assess the factorability of the UWES-9 data. Five indices were used to inspect model fit: (1) Mean Square Error of Approximation (RMSEA); (2) Standardised Root Mean Square Residual (SRMR); (3) chi-square test; (4) Comparative Fit index (CFI); and (5) Tucker-Lewis index (TLI). While these traditional fit indices are widely reported, their fixed cutoff values (e.g., RMSEA < 0.06, CFI > 0.95) have been criticized for poor generalizability, particularly because their performance is highly dependent on the specific characteristics of the model being tested^42^. Therefore, to provide a more rigorous and accurate assessment of model fit, we adopted the Dynamic Fit Index (DFI) approach^42,43^. This method generates customized fit index cutoffs tailored to the unique characteristics of each model, such as the number of factors, items, sample size, and the magnitude of factor loadings^42,43^. Using Monte Carlo simulation, the DFI method generates a distribution of fit indices for the researchers’ model under the assumption that it is correctly specified (Level-0) and compares it to distributions where the model contains a degree of hypothetical misspecification (e.g., Level-1, containing minor omitted cross-loadings). This process yields a direct, empirical benchmark (the Level-0 cutoff) representing the expected fit for a well-fitting model with the current data’s characteristics. The Weighted Least Squares Mean and Variance adjusted (WLSMV) on the polychoric correlation matrix was used to fit CFA models.

The invariance of a measurement was explored between males and females. Configural, metric, scalar, and strict invariance were supported if $\:\varDelta\:$ CFA was < 0.01 between invariance models^44^. The scale reliability was measured by the McDonald’s and also by the Cronbach’s. In addition to these, model-based Composite Reliability (CR) was calculated to assess the internal consistency of each factor, with values > 0.70 indicating good reliability^45^.

Construct validity was evaluated in several ways. First, we explored internal convergent validity during CFA, which assessed the degree to which items of a specific factor are related. More specifically, this was tested using the Average Variance Extracted (AVE), with values > 0.50 considered acceptable^45^. Next, we assessed, internal discriminant validity, which evaluated whether the factors are statistically distinct. For this purpose, the Heterotrait–Monotrait Ratio of Correlations (HTMT) test was used. If values from this test do not reach 0.90 discriminant validity is supported^46^. In the further step, we tested convergent and divergent validity externally i.e., via zero-order Spearman rank correlations with self-efficacy, neuroticism, and extraversion (convergent), and with spirituality (divergent). Relatedly, to provide a formal test of external discriminant validity, the magnitudes of the dependent correlations between the UWES-9 subscales and these external criteria were compared using the z-test from Hittner, May, and Silver^47^. To quantify the magnitude of these differences, we calculated Cohen’s q as an effect size. Cohen’s q values are interpreted as follows: q ≈ 0.10 indicates a small effect, q ≈ 0.30 a medium effect, and q ≈ 0.50 a large effect.

Due to the non-normal distribution of the data, an association between chronic health illnesses, health risk behavior, and UWES-9 was calculated using logistic regression. In the logistic models, the outcome variable was the presence of an individual chronic illness or the practice of health risk behavior. The UWES-9 score was set as a predictor. Education and work position were covariates. Both crude and adjusted effects were estimated. The p-values were corrected using the Bonferroni correction.

Comparisons between socio-demographic groups in the UWES-9 total and subscale scores were performed using the Kruskal–Wallis test. Significant omnibus tests were followed by post-hoc analysis using the Games–Howell test for groups with unequal variances or Dunn’s test where variances were equal. To quantify the magnitude of effects, epsilon-squared () was reported for the overall Kruskal–Wallis test, while Cohen’s *d* and the Rank–Biserial Correlation (*rbc*) were reported for the respective post-hoc comparisons. The interpretation of effect sizes followed established conventions: for epsilon-squared, values of 0.01, 0.06, and 0.14 are considered small, medium, and large effects, respectively. For Cohen’s *d*, benchmarks of 0.2, 0.5, and 0.8 were used for small, medium, and large effects, and for the rank-biserial correlation, values of 0.1, 0.3, and 0.5 were used to interpret small, medium, and large effects. All statistical calculations were conducted in R^48^. Primary packages used for analysis included: *lavaan*^49^, *papaja*^50^, *psych*^51^, *and usf*^52^.

## Results

### Sociodemographic results

Results of the Kruskal–Wallis, test followed by the Games–Howell and the Dunn test, revealed that there are significant differences in sociodemographic groups in the UWES-9 total and subscale scores: professional staff had significantly higher scores in the UWES-9 total, and vigor, absorption, and dedication subscale scores as compared with workers. Similarly, managers reported higher UWES-9 total scores and dedication and vigor subscale scores than workers (Table 1, 2). In terms of education, individuals with a higher vocational school or university degree had a significantly higher total UWES-9 score compared to those with non-graduation high school or lower education. For the absorption subscale, the university-educated group scored significantly higher than both the non-graduation high school and the high school educated groups (Tables 1, 2). Furthermore, a significant difference was found based on family status, where individuals in a relationship reported a higher score on the absorption subscale than those not in a relationship. There were no other significant differences between sociodemographic groups.

**Table 1:** Descriptive statistics of the sample by demographic variables (*N* = 648).

Variable	Category	Total	UWES-9 (total)	UWES_D	UWES_A	UWES_V
		n (%)	M (SD)	M (SD)	M (SD)	M (SD)
Gender	Male	406 (62.7)	39.02 (11.83)	13.21 (4.38)	13.11 (4.12)	12.70 (4.07)
	Female	242 (37.4)	40.80 (12.94)	13.66 (4.87)	13.70 (4.51)	13.45 (4.25)
Education	Basic school	19 (2.9)	33.68 (14.67)	11.05 (5.03)	11.68 (5.23)	10.95 (4.85)
	Non-graduation high school or lower	235 (36.3)	38.45 (13.16)	12.96 (4.81)	12.77 (4.68)	12.72 (4.42)
	High school	188 (29.0)	39.59 (12.32)	13.39 (4.50)	13.20 (4.30)	13.00 (4.21)
	Higher vocational school/University	206 (31.8)	41.73 (10.56)	14.06 (4.21)	14.23 (3.47)	13.43 (3.62)
Family status	Not in relationship	108 (16.7)	36.63 (12.07)	12.38 (4.46)	12.23 (4.34)	12.02 (4.05)
	In relationship	132 (20.4)	40.25 (11.79)	13.62 (4.37)	13.80 (4.11)	12.83 (4.10)
	Married	297 (45.8)	40.15 (11.98)	13.47 (4.53)	13.54 (4.12)	13.14 (4.03)
	Divorced	101 (15.6)	40.29 (13.52)	13.69 (4.95)	13.13 (4.64)	13.47 (4.52)
	Widow/Widower	10 (1.5)	45.50 (12.64)	15.20 (4.66)	14.80 (4.87)	15.50 (3.69)
Religiosity	Yes, I am a member of church	50 (7.7)	40.68 (11.10)	13.80 (4.44)	13.56 (3.86)	13.32 (3.64)
	Yes, but not a member of a church	131 (20.2)	38.59 (12.71)	12.98 (4.65)	13.09 (4.53)	12.52 (4.23)
	No	344 (53.1)	40.11 (12.20)	13.54 (4.50)	13.43 (4.23)	13.14 (4.14)
	No, convinced atheist	123 (19.0)	39.27 (12.51)	13.18 (4.74)	13.21 (4.32)	12.88 (4.29)
Work position	Worker	337 (52.0)	37.76 (13.12)	12.67 (4.84)	12.60 (4.54)	12.49 (4.44)
	Professional worker	227 (35.0)	41.28 (10.95)	14.00 (4.11)	14.01 (3.76)	13.26 (3.81)
	Chief worker	84 (13.0)	43.08 (10.85)	14.55 (4.17)	14.40 (4.04)	14.13 (3.53)

UWES-9, Utrecht Work Engagement Scale—9 items; D, Dedication; A, Absorption; V, Vigor; M, Mean; SD, Standard Deviation; n,  count. Percentages are rounded to one decimal place.

**Table 2:** Group comparisons of UWES-9 and subscales by selected demographic Variables.

Variable	Group comparison results
Education	UWES-9: H(3) = 10.47, *p* = .015, ε^2^ = 0.01 (Non-graduation high school or lower vs. Higher vocational school/University, t = 3.28, *p* = .021, d = − 0.27) UWES_D: H(3) = 9.97, *p* = .019, ε^2^ = 0.01 UWES_A: H(3) = 13.29, *p* = .004, ε^2^ = 0.02 (Non-graduation high school or lower vs. Higher vocational school/University, t = 1.46, *p* = .001, d = − 0.35; High school vs. Higher vocational school/University, t = 1.03, *p* = .047, d = − 0.27)
Family status	UWES-9: H(4) = 10.12, *p* = .038, ε^2^ = 0.01 UWES_A: H(4) = 10.51, *p* = .033, ε^2^ = 0.01 (Not in relationship vs. In relationship, z = 2.88, *p* = .040, r_bc = 0.19) UWES_V: H(4) = 11.62, *p* = .020, ε^2^ = 0.01
Work position	UWES-9: H(2) = 17.08, *p* < .001, ε^2^ = 0.02 (Worker vs. Professional worker, t = 3.52, *p* = .002, d = − 0.29; Worker vs. Chief worker, t = 5.32, *p* < .001, d = − 0.42) UWES_D: H(2) = 16.14, *p* < .001, ε^2^ = 0.02 (Worker vs. Professional worker, t = 1.33, *p* = .001, d = − 0.29; Worker vs. Chief worker, t = 1.88, *p* = .001, d = − 0.40) UWES_A: H(2) = 20.99, *p* < .001, ε^2^ = 0.03 (Worker vs. Professional worker, t = 1.41, *p* < .001, d = − 0.33; Worker vs. Chief worker, t = 1.80, *p* = .001, d = − 0.41) UWES_V: H(2) = 10.88, *p* = .004, ε^2^ = 0.01 (Worker vs. Chief worker, t = 1.64, *p* = .001, d = − 0.38)

*H* = Kruskal–Wallis test; *t* = t-test; *z* = z-score; *p* = p-value; d = Cohen’s d; *r_bc* = rank biserial correlation; *ε*^2^ = epsilon squared (effect size). Only significant group comparisons are presented. Group differences were assessed using the Kruskal-Wallis test, reported as the *H* statistic with its degrees of freedom (df), p-value, and epsilon-squared effect size. Significant omnibus tests were followed by pairwise post-hoc comparisons. For groups with unequal variances, the Games-Howell test is reported (t-statistic and Cohen’s *d* effect size). For groups with equal variances, Dunn’s test is reported (z-statistic and Rank-Biserial Correlation, *r_bc*, effect size). UWES-9 = Utrecht Work Engagement Scale—9 items; D = Dedication; A = Absorption; V = Vigor.

### Confirmatory factor analysis (CFA)

Bartlett test ($\:{\chi\:}^{2}$ (36) = 5565.42, *p* < .001), as well as the KMO index (0.96), revealed that UWES-9 data are sufficiently correlated to perform CFA.Next, a series of CFAs were conducted to determine the optimal factorial structure of the UWES-9 for the Czech sample. Results of these analyses are presented in Table 3. Initial models based on standard one-factor, two-factor, hierarchical, and the original correlated three-factor structures demonstrated poor fit to the data. For instance, the standard three-factor model yielded an RMSEA of 0.09, which was substantially higher than its dynamically-generated Level-0 cutoff of 0.03, indicating a significant degree of misfit. This led to the testing of more complex models that would be theoretically meaningful and, at the same time, account for sources of local misfit. Thus, in the next step, we tested bi-factor models.

Fitting bi-factor models, however, resulted in severe problems with estimation, making the results of these models not possible to interpret. Therefore, in the further step, we tested factor three models that reached the highest model fit in past studies. The first was the three-factor model, firstly proposed by Domínguez-Salas et al.^38^, which included correlated errors between items 1 and 2 (vigor) and items 8 and 9 (absorption). The second, a more complex model following Zecca et al.^53^, included these same two correlations between residuals plus a third between items 3 and 4 (dedication). The third and the most complex was the model of Balducci et al.^54^, who proposed the same correlations between error terms as Domínguez-Salas et al.^38^, with additional correlations between items 2 and 5 (vigor) and 6 and 8 (absorption).

It was revealed that all three of these models exhibit the best fit with the data as compared with other tested models. Thus, in the further step, we examined which of these three factor models provided the most optimal fit. The Chi-square difference test comparing these models revealed that the simpler Domínguez-Salas et al.^38^ model (χ^2^diff(2) = 13.82, p = *p* < .001) and model of Zecca et al.^53^ (χ^2^diff(1) = 0.04, *p* = .84) represented a statistically significant loss of fit compared to the more complex model of Balducci et al.^54^. Therefore, the Balducci et al.^54^ model can be considered as the best-fitting in our data.

**Fig. 2:**
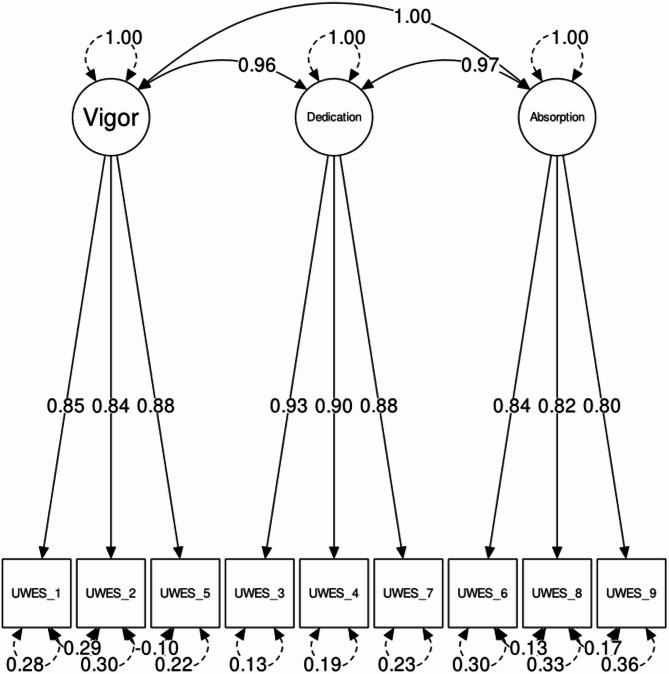
SEM plot of the UWES-9 three-factor solution with factor loadings and item residuals.

Further evaluation of this final model (see Fig. 2) revealed an excellent values of most of fit indices: χ^2^(20) = 158.853; *p* < .001; CFI = 0.987; TLI = 0.976; RMSEA = 0.077; SRMR = 0.017. However, its RMSEA was higher than its dynamically generated cutoff of 0.038, it still contains a non-trivial degree of misfit. This suggests that while this three-factor structure with modifications is the best representation of the data, the UWES-9 scale may not perfectly capture the construct of work engagement within the present study sample. Factor loadings (λ) in the Balducci et al.^54^ model were high (ranging from: 0.80 to 0.93) as were correlations between the three factors (see Fig. 1). Modification indices did not suggested high change in χ^2^ in case of releasing constrains between UWES-9 items. Correlation between residuals in manifest variables was low: r = range (− 0.04–0.04). Correlation matrix depicting relationships between item residuals can be found in the Supplementary Table 1.

**Table 3:** Empirical fit indices for competing CFA models of the UWES-9, supplemented with a dynamic fit indices (DFI) analysis of robustness to misspecification.

Misspecification	Model 1 One Factor Schaufeli				Model 2 Hierarchical Dominguez Salas				Model 3 Three Factor Standard Schaufeli			
	SRMR	RMSEA	CFI	Magnitude	SRMR	RMSEA	CFI	Magnitude	SRMR	RMSEA	CFI	Magnitude
Level-0	0.012	0.026	0.999		0.012	0.039	0.996	None	0.011	0.032	0.997	None
Level-1	0.013	0.036	0.999		None	None	None	0.177	None	None	None	0.177
Level-2	0.017	0.059	0.997		None	None	None	0.143	None	None	None	0.143
Level-3	0.022	0.09	0.994									
Fitted model	0.026	0.139	0.986		0.021	0.092	0.978		0.021	0.092	0.978	

Misspecification	Model 4a Two Factor Vigor Dedication Willmer				Model 4b Two Factor Vigor Absorption Chaudhary				Model 4c Two Factor Dedication Absorption Panthee			
	SRMR	RMSEA	CFI	Magnitude	SRMR	RMSEA	CFI	Magnitude	SRMR	RMSEA	CFI	Magnitude
Level-0	0.012	0.033	0.997	None	0.012	0.034	0.997	None	0.012	0.035	0.996	None
Level-1	None	None	NONE	0.174	None	None	None	0.187	None	None	None	0.19
Fitted model	0.024	0.106	0.968		0.022	0.094	0.974		0.024	0.107	0.967	

Misspecification	Model 5a Modified 3 F Balducci				Model 5b Modified 3 F Chaudhary				Model 5c Modified 3 F Dominguez			
	SRMR	RMSEA	CFI	Magnitude	SRMR	RMSEA	CFI	Magnitude	SRMR	RMSEA	CFI	Magnitude
Level-0	0.011	0.038	0.997	None	0.011	0.033	0.997	None	0.011	0.036	0.997	None
Level-1	None	None	None	0.122	None	None	None	0.178	None	None	None	0.15
Level-2					None	None	None	0.144	None	None	None	0.121
Fitted model	0.017	0.077	0.987		0.021	0.093	0.978		0.017	0.076	0.986	

Misspecification	Model 5d Modified 3 F Littman				Model 5e Modified 3 F Seppala				Model 5 g Modified 3 F Zecca			
	SRMR	RMSEA	CFI	Magnitude	SRMR	RMSEA	CFI	Magnitude	SRMR	RMSEA	CFI	Magnitude
Level-0	0.011	0.035	0.997	None	0.011	0.034	0.997	None	0.011	0.037	0.997	None
Level-1	None	None	None	0.178	None	None	None	0.15	None	None	None	0.15
Level-2	None	None	None	0.121	None	None	None	0.143	None	None	None	0.121
Fitted model	0.018	0.077	0.985		0.02	0.091	0.979		0.017	0.078	0.986	

SRMR, standardized root mean square residual; RMSEA , root mean square error of approximation; CFI, comparative fit index. Bold values indicate the fitted model results. None indicates parameter was not estimated for that specification level. Magnitude reflects the extent of model misspecification of the simulated error at each DFI threshold.

### Item statistics and reliability

Internal consistency of the UWES-9 total score was excellent: Cronbach’s $\:\alpha\:$ = 0.96, 95% CI[0.96–0.96] and McDonald’s $\:{\omega\:}_{t}$ = 0.96, 95% CI[0.96–0.96]. When assessing the internal consistency of the UWES subscales, the highest values were yielded by the dedication subscale: Cronbach’s $\:\alpha\:$ = 0.93 95% CI[0.92–0.94] and McDonald’s $\:{\omega\:}_{t}$ = 0.93 95% CI[0.92–0.94] followed by the vigor subscale: Cronbach’s $\:\alpha\:$ = 0.90 95% CI[0.89–0.91] and McDonald’s $\:{\omega\:}_{t}$ = 0.90 95% CI[0.89–0.91]. The lowest internal consistency was observed in the absorption factor: Cronbach’s $\:\alpha\:$ = 0.88, 95% CI[0.86–0.89] and McDonald’s $\:{\omega\:}_{t}$ = 0.88, 95% CI[0.86–0.89]. Reliability of the UWES-9 was supported by the CR: it´s values for vigor (0.86), dedication (0.91), and absorption (0.82), were excellent as they all range above the 0.70 threshold for good reliability. However, the omega hierarchical analysis ($\:{\omega\:}_{h}$= 0.92) revealed that most of the subscale reliability (73–89%) was attributable to the general factor rather than specific dimensions, with group-specific factors contributing only 5–11% of reliable variance. In contrast, the general engagement factor accounted for 90% of reliable variance, providing strong empirical support for using the UWES-9 as a total score. The Table 4 illustrates statistics of UWES-9 items. In general, correlations between these items and item-total correlations were high. The lowest item-total correlation had item 9.

**Table 4:** Item statistic and polychoric correlations between the UWES-9 items.

Items	UWES_1	UWES_2	UWES_3	UWES_4	UWES_5	UWES_6	UWES_7	UWES_8	UWES_9	ITC	Skewness	kurtosis	M (SD)
UWES_1	1									0.84	− 0.35	− 0.28	4.31 (1.48)
UWES_2	0.79***	1								0.81	− 0.43	− 0.34	4.56 (1.49)
UWES_3	0.75***	0.73***	1							0.89	− 0.3	− 0.56	4.43 (1.59)
UWES_4	0.73***	0.73***	0.84***	1						0.85	− 0.28	− 0.87	4.23 (1.71)
UWES_5	0.75***	0.71***	0.82***	0.76***	1					0.84	− 0.22	− 0.75	4.11 (1.65)
UWES_6	0.75***	0.72***	0.74***	0.7***	0.71***	1				0.81	− 0.6	− 0.22	4.76 (1.54)
UWES_7	0.7***	0.69***	0.82***	0.78***	0.77***	0.7***	1			0.83	− 0.43	− 0.6	4.72 (1.66)
UWES_8	0.71***	0.71***	0.73***	0.72***	0.68***	0.73***	0.69***	1		0.82	− 0.55	− 0.44	4.6 (1.63)
UWES_9	0.66***	0.65***	0.72***	0.73***	0.68***	0.67***	0.7***	0.72***	1	0.78	− 0.14	− 0.82	3.96 (1.66)

*p < .05; ** *p* < .01; *** *p* < .001, *M* = Mean, *SD* = Standard Deviation, ITC = Item-total correlation corrected for scale reliability and item overlap. All coefficients are polychoric correlations.

### Construct validity evaluation

With the best-fitting model established, we conducted an evaluation of its construct validity. We first assessed convergent validity by calculating the AVE for each factor. The results for vigor (0.73), dedication (0.82), and absorption (0.67) all exceeded the 0.50 treshold, indicating that for each factor, a majority of the variance in its items was captured by the construct itself rather than by measurement error.

However, given the high inter-factor correlations (see Fig. 2), a specific test of discriminant validity was necessary to determine if the factors were empirically distinct. Thus, we employed the HTMT for this purpose. The analysis yielded values of 0.95 (vigor-dedication), 0.96 (vigor-absorption), and 0.94 (dedication-absorption). As all values substantially exceeded the 0.90 threshold, this provided definitive evidence that the factors, while structurally sound, are not internally distinct from one another.

This finding led to our final validity test, where we examined the scale’s relationships with external criteria to see if the subscales behaved differently despite their internal overlap. As shown in Table 5, the UWES-9 scores behaved as expected: the total score and all subscales were positively associated with extraversion and self-efficacy, and negatively associated with neuroticism. For divergent validity, no significant correlation was found with spirituality, with the exception of the dedication subscale.

To formally test whether the magnitude of these correlations differed across subscales, we used the z-test and calculated Cohen’s q to quantify the effect size of the differences. The analysis revealed specific differences in the subscales’ external relationships. For example, the correlation between vigor and extraversion was significantly stronger than that of absorption and extraversion, representing a small effect (q = 0.13, *p* < .001). In contrast, the difference in their correlations with neuroticism was not always statistically significant; the difference between vigor and dedication was negligible (q = 0.03, *p* = .132), while the difference between vigor and absorption was significant with a small effect (q = 0.11, *p* < .001). Taken together, despite a high overlap between UWES-9 dimensions, subscales of the UWES-9 exhibit some statistically distinct relationships with external criteria.

Correlation analysis indicated that there is significant positive association between all UWES-9 subscale and total score and extraversion. The highest correlation was found in the vigor subscale. In addition, there was significant negative correlation between all UWES-9 subscales and total score with neuroticism. The highest association was also found in the vigor subscale. Moreover, the UWES-9 total and its all subscales were associated with self-efficacy. The strongest association was observed in the vigor subscale. Finally, there was no correlation between the UWES-9 composite and subscale score with spirituality with exception of dedication subscale (see Table 5).

**Table 5:** Correlation matrix of the UWES-9, personality characteristics, and sociodemographic indicators.

	1	2	3	4	5	6	7	8	9	M(SD)
1. UWES-9	–									39.69 (12.27)
2. UWES V	0.94***	–								12.98 (4.15)
3. UWES D	0.95***	0.85***	–							13.38 (4.57)
4. UWES A	0.93***	0.82***	0.83***	–						13.33 (4.28)
5. BFI E	0.19***	0.23***	0.18***	0.13***	–					24.20 (5.21)
6. BFI N	− 0.19***	− 0.23***	− 0.18***	− 0.12**	− 0.27***	–				23.02 (5.70)
7. Age	0.03	0.06	0.01	0.02	− 0.01	− 0.10**	–			43.65 (10.08)
8. Gender	0.07	0.08	0.05	0.07	0.06	0.20***	0.08*	–		1.38 (0.49)
9. DSES	0.13	0.09	0.17*	0.11	0.09	− 0.06	− 0.02	0.10	–	2.39 (1.10)
10. GSES	0.28***	0.30***	0.26***	0.25***	0.31***	− 0.44***	0.08*	− 0.09*	0.13	28.43 (4.95)

*p < .05; ** *p* < .01; *** *p* < .001; *SD* = standard deviation, *M* = mean, UWES-9, Utrecht Work Engagement Scale; BFI N, Big Five Inventory-Neuroticism subscale; BFI E, Big Five Inventory-Extraversion subscale; UWES A, Utrecht Work Engagement Scale-Absorption subscale; UWES D, Utrecht Work Engagement Scale-Dedication subscale; UWES V, Utrecht Work Engagement Scale-Vigor subscale; DSES, Daily Spiritual Experience Scale; GSES, General Self-Efficacy Scale.

### Invariance testing and factor loadings

The results of the measurement invariance testing are summarized in Table 6. In this analysis, we report the scaled fit indices, as the robust versions could not be computed for the scalar and strict invariance models. It was revealed that in female group, factor intercorrelations exceeded 1.0 (vigor-absorption *r* = 1.02). To ensure model stability, correlated residuals from the original Italian validation were removed, as they caused identification issues in the smaller female subsample. After this removal, the model included correlated errors only between items 1 and 2 (vigor) and items 8 and 9 (absorption). After this modification the model was the same as the Domínguez-Salas et al.^38^ model. In this simplified three-factor model, the change in the scaled Comparative Fit Index (CFI) was less than 0.01 across all model comparisons. This finding strongly supports full measurement invariance and indicates that the UWES-9 assesses work engagement equivalently in males and females.

**Table 6:** Measurement equivalence of the UWES-9 between genders.

Model	χ^2^	df	*p*-value	CFI	TLI	RMSEA	SRMR
Baseline	168.916	22	*p* < .001	0.994	0.99	0.102 90% CI (0.088–0.116)	0.017
Configural	216.885	44	*p* < .001	0.993	0.988	0.11 90% CI (0.096–0.125)	0.02
Metric	174.424	50	*p* < .001	0.995	0.993	0.088 90% CI (0.074–0.102)	0.022
Scalar	187.198	92	*p* < .001	0.996	0.997	0.057 90% CI (0.045–0.068)	0.021
Strict	187.198	92	*p* < .001	0.996	0.997	0.057 90% CI (0.045–0.068)	0.021

χ^2^, chi-square; *df*, degrees of freedom; CFI, Comparative Fit Index; TLI , Tucker–Lewis Index; RMSEA, Root Mean Square Error of Approximation; SRMR, Standardized Root Mean Square Residual; CI, confidence interval.

### Association of the UWES-9 with chronic health illnesses

Results of the regression analysis revealed that work engagement is significantly related with chronic diseases. Specifically, higher work engagement was significantly related with lower probability of developing skin diseases or eczema (in crude effect) pain of unclear origin (both crude and adjusted effect), (see Table 7).

**Table 7:** Logistic regression table depicting associations (in odds ratios) between the UWES-9 and chronic diseases.

	Skin diseases eczema	Pain of unclear origin	Hypertension	Diabetes	Arthritis
Crude effect	0.98* (0.95, 1.00)	0.93** (0.89, 0.97)	1.01 (0.99, 1.03)	1.00 (0.98, 1.03)	0.97 (0.95, 1.01)
Adjusted effect	0.98 (0.96, 1.00)	0.94** (0.90, 0.98)	1.01 (0.99, 1.03)	1.01 (0.98, 1.03)	0.98 (0.95, 1.01)
	Depression/Anxiety	Migraine	Cancer	Thyroid disease	Astma
Crude effect	0.99 (0.96, 1.02)	1.00 (0.97, 1.03)	1.00 (0.95, 1.07)	1.01 (0.99, 1.04)	0.98 (0.96, 1.00)
Adjusted effect	1.00 (0.97, 1.02)	1.00 (0.97, 1.04)	1.00 (0.94, 1.07)	1.02 (1.00, 1.05)	0.98 (0.96, 1.01)
	Gastric or duodenal ulcers	Chronic lung disease	Skin diseases eczema	Allergy	Pain in the small pelvis
Crude effect	1.01 (0.95, 1.10)	0.97 (0.92, 1.02)	0.98* (0.95, 1.00)	0.99 (0.97, 1.01)	1.00 (0.96, 1.05)
Adjusted effect	1.01 (0.94, 1.10)	0.97 (0.93, 1.02)	0.98 (0.96, 1.00)	0.99 (0.97, 1.01)	1.01 (0.97, 1.05)
	Ischemic heart disease	Obesity	Stroke	Back pain	
Crude effect	1.00 (0.93, 1.08)	0.99 (0.97, 1.01)	0.95 (0.87, 1.04)	0.99 (0.97, 1.00)	
Adjusted effect	0.99 (0.92, 1.07)	0.99 (0.97, 1.01)	0.95 (0.86, 1.04)	0.99 (0.98, 1.01)	

*p < .05; ** *p* < .01; *** *p* < .001; results are reported as odds ratios. Education and work position were included as covariates in the adjusted effects. Values in parentheses represent 95% confidence intervals for the odds ratios.

### Association of the UWES-9 with health risk behaviour

Results of logistic regression suggested that there is no relationship between work engagement and the smoking, alcohol drinking, drug abuse, coffee drinking or using computer or television for recreation in both crude and adjusted effect (Supplementary Table 2). Variable smoking was the closest to the significance threshold.

## Discussion

This study aimed to examine the psychometric properties of the Czech version of the UWES-9 by exploring its factor structure, convergent and divergent validity, and internal consistency, as well as to test for measurement equivalence. Results revealed that the three-factor solution yielded the best fit with the data. Results also demonstrated the UWES-9’s excellent internal consistency. Its validity was supported by a negative association with neuroticism, a positive association with self-efficacy and extraversion, and by the absence of a significant relationship with spirituality. A higher UWES-9 total score was found in professional staff, managers, and people with vocational school or university education. Measurement equivalence testing confirmed that the UWES-9 measures WE consistently across genders. Finally, a higher UWES-9 score predicted a lower probability of developing skin diseases or eczema and pain of unclear origin.

### Dimensionality

Hypothesis, that a three-factor solution would best fit the data, received partial support. CFA results showed that, among the tested models, the modified three-factor structure proposed by Balducci et al.^54^ demonstrated the best overall fit in the Czech sample when compared to alternative one- and two-factor solutions. However, although most fit indices reached excellent values, the RMSEA was above its cutoff, indicating a remaining degree of model misfit. This suggests that while the three-factor model with correlated residuals provides the most adequate representation of the data, it does not perfectly capture the dimensionality of the UWES-9 in the Czech context. In contrast, some validation studies from a slightly different cultural settings reported acceptable RMSEA values for similar modified three-factor solutions^38,53^, which highlights potential cultural variability in the factorial performance of the UWES-9.

### Internal consistency

The lowest internal consistency was observed in the absorption factor. This aligns with other studies^10^, indicating the lowest internal consistency in this subscale. One of the reasons for the lowest internal consistency of this subscale might be caused by item 9 (“I get carried away when I’m working”), which had the lowest item-to-item correlation and the lowest item-total correlation. This item contains a metaphor that is not unequivocally positive and thus could be misunderstood or misinterpreted by some respondents. Future studies should review the translation of the phrase to improve understanding within the Czech language’s cultural environment. However, the factor load on item 9 was 0.82, which can still be considered acceptable and a meaningful contribution to the scale.

### Measurement invariance

Measurement equivalence testing suggested that the UWES-9 assesses WE invariantly between males and females. This finding aligns with results from other studies^55^. Invariance was tested on four levels: configure, metric, scalar, and strict. As the UWES-9 was found to be invariant in each of these four levels, it can be recommended that this measure be used to assess gender differences in the UWES-9 in different research settings. If significant differences between genders are found, it can be assumed that this difference is linked to the true differences in the latent variable instead of differences in how males and females respond to the UWES-9 questions. In summary, this finding strengthens the validity of the Czech UWES-9 for gender-based comparisons in research and practice.

Our findings must be interpreted in light of substantial inconsistencies reported in the literature regarding the UWES-9’s factorial structure and cross-cultural measurement invariance^38,40^. A systematic review of 21 studies revealed ambiguous results: six favoured the original three-factor model, six supported a one-factor structure, and eight concluded that both were acceptable; one study confirmed neither^21^. Even where the three-factor model fit best (e.g., Italy, Norway, Nepal, France), fit indices were not always optimal, and modifications were often needed^53,54,56,57^. In Japan and Brazil, a one-factor model predominated^17,58^, while studies in Russia, Finland, Sweden, and Vietnam found both structures acceptable, often with very high inter-factor correlations (*r* > .90), suggesting that engagement can function as a unidimensional construct in some contexts^10,21,55,59^.

Measurement invariance testing has yielded similarly inconsistent results^60,61^. For example, a cross-cultural study of teachers from Western (Australia, Canada) and non-Western (China, Indonesia, Oman) settings failed to establish invariance across the broader cultural categories, although partial invariance was observed within each subgroup^61^. In an Italian–Dutch comparison, scalar invariance was not achieved, preventing meaningful mean-level comparisons^54^. Even when configural invariance holds in many studies, factor loadings often differ systematically across countries^60–62^. These findings may reflect both translation and cultural adaptation issues and deeper conceptual differences in how engagement is experienced and expressed^8^. Dimensions like vigor may be differently valued in collectivistic versus individualistic cultures, while modesty bias in East Asia may systematically lower self-reported scores^10,61^. Further cross-cultural studies with carefully matched occupational groups, equivalent translations and adequate sample sizes are needed to determine whether the UWES-9 can support valid international comparisons or is better understood as a context-specific measure.

### Convergent validity: extraversion, neuroticism, and self-efficacy

This study found a positive correlation between the UWES-9 total score and extraversion (supporting H2a), a negative correlation between the UWES-9 total score and neuroticism (supporting H2b), and a positive association between self-efficacy and UWES-9 total score (supporting H3). This aligns with results from other studies in which these associations were reported^23,62^. Consistent with previous findings^5,63^, the UWES-9 total and all its subscales were positively associated with self-efficacy. These results support the convergent validity of the instrument because, based on assumptions of previous researchers investigating WE, these two constructs should be significantly associated^62,64^.

While testing divergent validity, correlation analysis between the UWES-9 and spirituality showed no significant correlation with total UWES-9, but surprisingly, there was a correlation with the dedication subscale. Although contradictory to our expectations, these results align with the study of Ashmos and Duchon^65^. However, it is essential to note that the conceptualisation of spirituality differed significantly from this study. The concept of Ashmos and Duchon^65^ sees spirituality as an employee’s inner life with a sense of purpose and meaning, and a sense of being interconnected with workmates. This differs from the concept and measurement used in our study, where spirituality means experiencing a connection with the transcendent in daily life. In the context of the present study, the positive link between work dedication and spirituality can be explained as follows: dedication, which involves a sense of meaning and enthusiasm toward work, may overlap with intrinsic aspects of spirituality, such as purpose and fulfilment.

### Convergent validity: job level and age

In line with Hypothesis 4, we found a higher score in the UWES-9 total and its subscale scores for professional staff and managers (except absorption) than for workers. Our finding, that blue-collar workers are less engaged than managers, matches the theoretical assumptions of Schaufeli et al.^5^, maybe because they draw less on job resources, which are known to be positively related to WE.

There was no significant correlation between the UWES-9 total and the UWES-9 subscales and age. This contradicts our expectations (Hypothesis 4) and findings from previous studies^5,53,66^. One possible explanation is that the relationship between age and WE becomes more pronounced in older age groups. Studies focusing on participants over 50 years old have found significant age-related effects on UWES scores^67^, suggesting that age-related differences in WE may emerge later in life.

The current findings reveal a significant protective association between work engagement and specific chronic health conditions, particularly skin diseases/eczema and non-specific pain^17,68^. These results align with established theoretical frameworks and empirical evidence regarding the health-protective effects of work engagement^69–71^.

### Work engagement and skin health

The negative association between work engagement and skin diseases/eczema is consistent with extensive research documenting the “brain-skin connection” and the role of psychological stress in dermatological conditions^72,73^. Stress has been identified as a significant trigger or exacerbating factor in numerous skin conditions, including atopic dermatitis, psoriasis, urticaria, and eczema^74^. The skin possesses a fully functional peripheral equivalent of the hypothalamic–pituitary–adrenal (HPA) axis, making it particularly susceptible to stress-induced pathophysiological changes^72^. Work engagement may protect against skin diseases through multiple mechanisms. First, highly engaged employees typically experience lower levels of chronic psychological stress, which directly impacts the neuroimmunological pathways involved in skin inflammation^75^. Second, engagement is associated with better overall health behaviors and coping strategies, which may indirectly support skin health maintenance.

### Work engagement and pain management

The protective effect of work engagement against non-specific pain aligns with occupational health research demonstrating the health-promoting effects of positive work experiences^76^. Pain, particularly chronic and non-specific pain, is frequently associated with psychological stress, poor work conditions, and reduced quality of life. The Job Demand-Resources (JD-R) model suggests that engagement functions as a personal resource that buffers against various stressors and their associated health consequences^77^. Engaged employees may experience reduced pain through enhanced resilience, improved stress management, and potentially altered pain perception and coping mechanisms. Notably, the persistence of these associations even after controlling for covariates^55,78,79^ suggests that engagement may exert direct protective effects beyond its correlations with other positive factors.

### Strengths and limitations

The present study demonstrates several methodological strengths, notably the use of a large and diverse sample from various occupational backgrounds, which enhances the generalizability of the findings within the Czech context. A major strength of this study lies in its rigorous data quality control, including the careful screening and exclusion of problematic observations such as inconsistencies in participants’ responses and unusually rapid completion times, which increases the validity of our results.

The limitations include the non-probabilistic sampling method. Although the Czech National Panel maintains a registry of over 40,000 respondents from which it selects a sample with representative characteristics using demographic quotas, the recruitment process does not follow random selection procedures. Consequently, since the sample is not drawn through a probabilistic design, statistical generalization to the broader population may be limited. Results suggest that translation was adequate because the psychometric parameters of the scale were good. This indicates that items were understood well. Another potential limitation is that the study was based on self-reported methods. Thus, we cannot deny the possibility that social desirability bias did not influence the results. Finally, the study sample was imbalanced in terms of gender.

### Implications for further research and practice

In terms of implications for research, given the stable dimensionality demonstrated in this study, future research should investigate the stability of the three-factor structure of the UWES-9 across different occupational sectors and demographic groups within the Czech context. Longitudinal validation studies are particularly warranted to establish the temporal stability of the UWES-9 and examine its predictive validity for various health and organizational outcomes over time. Such studies would strengthen the evidence base for using UWES-9 as both a diagnostic and monitoring tool in occupational settings. Additionally, longitudinal studies are warranted to examine the predictive validity of work engagement for various health outcomes. Future research should also explore cross-cultural validation studies comparing the Czech version with other European adaptations to provide insights into cultural factors influencing work engagement measurement.

Regarding practical implications of the present study, according to Eurostat data from May 2025, the unemployment rate in the Czech Republic was the lowest within the EU in December 2024. Specifically, it was 2.8% vs. 5.8% of the EU total^80^. In this context, where recruiting new employees is particularly challenging, retaining current staff becomes essential for organizations. Since higher WE is linked to lower turnover, the availability of a validated tool to measure WE, such as the UWES-9 presented in this study, is especially valuable. In such an environment, UWES-9 can be used to measure WE. This would provide a deeper understanding of WE and could be a base for creating targeted actions to increase WE. As a result, it would not only be a competitive advantage for employers but also contribute to the quality of life for employees.

For human resources (HR) professionals and organizational leaders, the validated Czech UWES-9 offers several concrete applications in talent management and organizational development. Engagement metrics can be integrated into performance management systems alongside other performance indicators. Organizations can focus on the overall UWES-9 score to guide interventions aimed at enhancing work engagement. Regular UWES-9 assessments can serve as an early warning system to identify employees at risk of disengagement, enabling proactive intervention before turnover occurs. Furthermore, UWES-9 data can inform the design of compensation and benefits packages by evaluating which organizational policies most effectively support employee engagement and well-being. The observed association between higher work engagement and reduced prevalence of certain chronic health conditions suggests that fostering engagement may yield tangible (though modest) health benefits, underscoring the potential benefit of integrating engagement strategies into broader occupational health programs.

## Conclusion

Summing up, the results showed that the Czech version of UWES-9 has good psychometric properties regarding reliability, factor structure, construct validity, and measurement invariance. We recommend scoring the UWES-9 as a composite score. The study findings prove that UWES-9 is a valid and reliable instrument to measure WE in the Czech cultural context.

## Supplementary Information

Below is the link to the electronic supplementary material.

Supplementary Material 1

Supplementary Material 2

Supplementary Material 3

## Data Availability

Data and study code used for statistical analysis of the present study are publicly available on the Open Science Framework (OSF) under the following link: [https://osf.io/w5anh/].
