# Supplementary material for: Validation of the Utrecht work engagement scale (UWES-9) in the Czech Republic
Source: Sci Rep. 2025 Nov 28;15:42767. doi: 10.1038/s41598-025-26907-z (PMC12663390; doi:10.1038/s41598-025-26907-z)
Supplement: Supplementary file 1 — Supplementary Material 1 [file 41598_2025_26907_MOESM1_ESM.pdf]

Validation of the Utrecht Work Engagement Scale (UWES-9) in the Czech Republic

Hypothesis Justification

Martin Heveri[1]*, Lukas Novak[1], Iva Polackova Solcova[1,2], Peter Tavel[1]

[1]Olomouc University Social Health Institute, Palacky University in Olomouc, Czech Republic

[2]The Czech Academy of Sciences, Institute of Psychology, Prague, Czech Republic

*** Correspondence:**

Martin Heveri, Univerzitni 244/22, 771 11, Olomouc, Czech Republic

martin.heveri@oushi.upol.cz

In this study, we focus on psychometric properties, especially on the reliability and validity of the Czech version of the UWES-9, which, to the best of our knowledge, has not been examined in previous studies. Specifically, we examined the internal consistency, factorial validity, convergent validity, and measurement invariance. We also explored differences in WE according to main sociodemographic characteristics and correlation with self-efficacy, neuroticism, and extraversion. Discriminant validity was tested by relationship to spirituality. Lastly, we examined the association of the UWES with chronic health illnesses and health risk behaviours. Based on the findings of previous research and theoretical assumptions, we set the following hypothesis:

Hypothesis 1: A three-factor solution of WE would best fit the data.

Hypothesis 2: The WE would be related to high levels of extraversion (H2a) and low levels of neuroticism (H2b).

Justification: Work engagement (WE) has been consistently linked to personality traits, particularly within the Five-Factor Model (FFM) framework. Research suggests that individuals with high levels of extraversion tend to experience greater work engagement, while those with high neuroticism demonstrate lower levels of engagement. Fukuzaki and Iwata [1] examined the relationship between personality dimensions and WE, revealing that extraversion exhibited a strong positive correlation with WE ($\rho = 0.38$). In contrast, neuroticism was negatively associated ($\rho = -0.36$). Their findings indicate that personality traits explain a significant proportion of variance in work engagement ($R^2 = 0.33$), supporting the notion that extraverted individuals, characterised by sociability, assertiveness, and positive affectivity, are more likely to engage with their work. Conversely, individuals with high neuroticism, marked by emotional instability and susceptibility to stress, tend to exhibit lower work engagement.

Similarly, Ansari [2] investigated the relationship between personality traits and employee engagement in a sample of IT professionals, employing a structural equation modelling (SEM) approach. The study confirmed that extraversion and neuroticism significantly influenced work engagement, further reinforcing that personality-related dispositions are crucial in shaping employees' engagement levels. Given these empirical findings, it is reasonable to expect that work engagement will be positively associated with extraversion (H2a) and negatively associated with neuroticism (H2b).

Hypothesis 3: There would be a significant positive association between self-efficacy and UWES-9 total score.

Justification: Self-efficacy, an individual's belief in their capacity to perform tasks successfully, has been recognised as a key psychological factor influencing work engagement. Empirical studies suggest that higher levels of self-efficacy are associated with greater engagement at work, as individuals with strong self-efficacy tend to demonstrate resilience, motivation, and proactive behavior in professional settings.

Bernales-Turpo et al. [3] examined the mediating role of work engagement in the relationship between job burnout, professional self-efficacy, life satisfaction, and job performance among Peruvian healthcare workers. Their structural equation modelling (SEM) analysis revealed that self-efficacy ($\beta = 0.557$, $p < 0.001$) was a significant positive predictor of work engagement. These findings suggest that individuals with higher self-efficacy are more likely to be engaged in their work, reinforcing the theoretical expectation that self-efficacious employees experience greater motivation and commitment.

Similarly, Zarrin et al. [4] explored the relationship between self-efficacy, reflective practice, and work engagement among nurses. Their findings indicated a significant positive association between self-efficacy and work engagement, with self-efficacious nurses demonstrating higher professional commitment and engagement levels. The study highlights the role of self-efficacy as a crucial psychological resource that fosters engagement in demanding work environments.

Given these empirical findings, self-efficacy is expected to be significantly and positively associated with the total Utrecht Work Engagement Scale (UWES-9) score, supporting the formulation of Hypothesis 3.

Hypothesis 4: The UWES-9 and its subscales would show positive weak correlations with age and managerial work positions.

Justification: Work engagement (WE) has exhibited a weak but positive correlation with age and managerial work positions. Research suggests that individuals tend to develop better emotional regulation, resilience, and coping strategies as they age, contributing to higher engagement levels [5]. A systematic review of 50 studies on older workers confirmed that work engagement tends to increase with age, primarily due to these emotional and psychological adaptations. Similarly, Zacher and Schmitt [6] reported a modest positive correlation between age and work engagement, suggesting that older employees may derive greater meaning and satisfaction from their work, leading to sustained or enhanced engagement.

The relationship between managerial roles and work engagement has also been explored in organisational research. Lu et al. [7] examined work engagement differences between supervisors and line-level employees and found that managerial positions were associated with significantly higher engagement levels. This can be attributed to increased autonomy, greater decision-making responsibilities, and access to job resources, such as professional recognition and personal development opportunities. Individuals in managerial roles may also experience higher intrinsic motivation, a key driver of work engagement.

While these relationships exist, the correlations between work engagement, age, and managerial positions are generally weak. Various moderating factors, such as job demands, personality traits, and organisational culture, may influence the strength of these associations [6]. Additionally, research suggests that the importance of specific work characteristics differs among age groups, indicating that job resources may affect engagement depending on an individual's career stage [5].

Given these findings, it is expected that the Utrecht Work Engagement Scale (UWES-9) and its subscales will exhibit weak but positive correlations with both age and managerial work positions, supporting the formulation of Hypothesis 4.

Referencies

1. Fukuzaki, T. & Iwata, N. Association between the five-factor model of personality and work engagement: a meta-analysis. *Ind. Health* **60,** 154–163 (2021).
2. Ansari, J. A. N. Driving Employee Engagement Through Five Personality Traits: An Exploratory Study. *Metamorph. J. Manag. Res.* **19,** 94–105 (2020).
3. Bernales-Turpo, D. *et al.* Burnout, Professional Self-Efficacy, and Life Satisfaction as Predictors of Job Performance in Health Care Workers: The Mediating Role of Work Engagement. *J. Prim. Care Community Health* **13,** 21501319221101845 (2022).
4. Zarrin, L., Ghafourifard, M. & Sheikhalipour, Z. Relationship between Nurses Reflection, Self-efficacy and Work Engagement: A Multicenter Study. *J. Caring Sci.* **12,** 155–162 (2023).
5. Mori, K. *et al.* Work engagement among older workers: a systematic review. *J. Occup. Health* **66,** uiad008 (2024).
6. Zacher, H. & Schmitt, A. Work Characteristics and Occupational Well-Being: The Role of Age. *Front. Psychol.* **7,** (2016).
7. Lu, L., Lu, A. C. C., Gursoy, D. & Neale, N. R. Work engagement, job satisfaction, and turnover intentions: A comparison between supervisors and line-level employees. *Int. J. Contemp. Hosp. Manag.* **28,** 737–761 (2016).
