# Supplementary material for: Validation of the Utrecht work engagement scale (UWES-9) in the Czech Republic
Source: Sci Rep. 2025 Nov 28;15:42767. doi: 10.1038/s41598-025-26907-z (PMC12663390; doi:10.1038/s41598-025-26907-z)
Supplement: Supplementary file 2 — Supplementary Material 2 [file 41598_2025_26907_MOESM2_ESM.docx]

**Supplementary Table 2 Logistic regression table depicting associations (in odds ratios) between the UWES and health risk behaviors.**

|  | **Smoked** | **Drunk alcohol** | **Used illegal drugs** | **Drunk coffee** | **Used television or computer for recreation** |  |
| --- | --- | --- | --- | --- | --- | --- |
| Crude effect | 1.00 (0.99, 1.02) | 1.00 (0.98, 1.01) | 0.97 (0.92, 1.02) | 1.01 (1.0, 1.02) | 1.01 (0.99, 1.03) |  |
| Adjusted effect | 1.01 (1.00, 1.03) | 1.00 (0.98, 1.01) | 0.98 (0.93, 1.04) | 1.01 (1.00, 1.03) | 1.01 (0.99, 1.03) |  |

*Note.* * p < 0.05; ** p < 0.01; *** p < 0.001, results are reported in odds ratios; Education and Work position were covariates in adjusted effect; values in brackets refer to 95% confidence interval for odds ratios
