# Supplementary material for: Validation of the Utrecht work engagement scale (UWES-9) in the Czech Republic
Source: Sci Rep. 2025 Nov 28;15:42767. doi: 10.1038/s41598-025-26907-z (PMC12663390; doi:10.1038/s41598-025-26907-z)
Supplement: Supplementary file 3 — Supplementary Material 3 [file 41598_2025_26907_MOESM3_ESM.docx]

**Supplementary Table 1 Lower-triangular correlations between residuals of the final factor solution.**

|  | UWES_1 | UWES_2 | UWES_5 | UWES_3 | UWES_4 | UWES_7 | UWES_6 | UWES_8 | UWES_9 |
| --- | --- | --- | --- | --- | --- | --- | --- | --- | --- |
| UWES_2 | 0 |  |  |  |  |  |  |  |  |
| UWES_5 | 0.01 | -0.01 |  |  |  |  |  |  |  |
| UWES_3 | -0.01 | -0.02 | 0.03 |  |  |  |  |  |  |
| UWES_4 | -0.01 | 0.01 | -0.01 | 0 |  |  |  |  |  |
| UWES_7 | -0.02 | -0.01 | 0.02 | 0 | -0.01 |  |  |  |  |
| UWES_6 | 0.04 | 0.03 | -0.03 | -0.02 | -0.02 | -0.01 |  |  |  |
| UWES_8 | 0.01 | 0.02 | -0.05 | -0.01 | 0.01 | -0.01 | 0.01 |  |  |
| UWES_9 | -0.01 | -0.02 | -0.02 | 0 | 0.03 | 0.02 | -0.02 | 0 |  |

*Note.* UWES = Utrecht Work Engagement Scale
